# Supplementary material for: Gut microbial factors predict disease severity in a mouse model of multiple sclerosis
Source: Nat Microbiol. 2024 Jul 15;9(9):2244–61. doi: 10.1038/s41564-024-01761-3 (PMC11371644; doi:10.1038/s41564-024-01761-3)
Supplement: Supplementary file 1 — Reporting Summary [file 41564_2024_1761_MOESM1_ESM.pdf]

nature portfolio

Corresponding author(s): Mahesh S. Desai

Last updated by author(s): Jun 30, 2024

Reporting Summary

Nature Portfolio wishes to improve the reproducibility of the work that we publish. This form provides structure for consistency and transparency in reporting. For further information on Nature Portfolio policies, see our Editorial Policies and the Editorial Policy Checklist.

Statistics

For all statistical analyses, confirm that the following items are present in the figure legend, table legend, main text, or Methods section.

n/a	Confirmed	
☐	☒	The exact sample size (*n*) for each experimental group/condition, given as a discrete number and unit of measurement
☐	☒	A statement on whether measurements were taken from distinct samples or whether the same sample was measured repeatedly
☐	☒	The statistical test(s) used AND whether they are one- or two-sided<br>*Only common tests should be described solely by name; describe more complex techniques in the Methods section.*
☐	☒	A description of all covariates tested
☐	☒	A description of any assumptions or corrections, such as tests of normality and adjustment for multiple comparisons
☐	☒	A full description of the statistical parameters including central tendency (e.g. means) or other basic estimates (e.g. regression coefficient) AND variation (e.g. standard deviation) or associated estimates of uncertainty (e.g. confidence intervals)
☐	☒	For null hypothesis testing, the test statistic (e.g. *F*, *t*, *r*) with confidence intervals, effect sizes, degrees of freedom and *P* value noted<br>*Give P values as exact values whenever suitable.*
☒	☐	For Bayesian analysis, information on the choice of priors and Markov chain Monte Carlo settings
☒	☐	For hierarchical and complex designs, identification of the appropriate level for tests and full reporting of outcomes
☐	☒	Estimates of effect sizes (e.g. Cohen's *d*, Pearson's *r*), indicating how they were calculated

Our web collection on statistics for biologists contains articles on many of the points above.

Software and code

Policy information about availability of computer code

Data collection

No custom software was used for data collection.

Data analysis

No custom algorithms or software were used for data analysis. The analysis pipelines in this study employ tools that have been previously described in published literature and are described below.

All figures and graphs were created using R Studio (Version 4.2.1) and the package ggplot2 (Version 3.3.6). Subsequent figure modifications were performed using Inkscape.

All data analyses and statistical computations were performed using R Studio (Version 4.2.1) using existing packages and functions. Correlations were calculated using the rcorr package (Version 0.4.4). Linear and nonlinear regressions were calculated using the drc package (Version 3.0.1). One-way ANOVA, Kruskal-Wallis test, t-tests and Wilcoxon rank-sum-tests were calculated using rstatix package (Version 0.7.0). Normality distribution of data sets before statistical computation was determined by applying a Shapiro-Wilk test. Outliers were determined using the outlier package (Version 0.15) and, importantly, confirmed using a Dixon test.

Construction of the phylogenetic tree of SM14 constituent strains
The phylogenetic tree was constructed based on full-length 16S rRNA gene sequences and analysed with Geneious Prime version 2021.2.2. A neighbour-joining tree build model was created using global alignment with free end and gaps, 65% similarity index cost matrix, and a Tamura-Nei genetic distance model.

V4 16S rRNA gene sequencing analysis:
The program mothur (v1.44.3) (DOI: https://doi.org/10.1128/AEM.01541-09) was used to process the reads according to the MiSeq SOP. For

gnotobiotic samples, taxonomy was assigned using a k-nearest neighbor consensus approach against a custom reference database corresponding to the SM14 taxa and potential contaminants (Citrobacter rodentium, Lactococcus lactis subsp. cremoris, Staphylococcus aureus, and Staphylococcus epidermidis). For SPF samples, taxonomy was assigned using the Wang approach against the SILVA v132 database. Count data was normalized by computing relative abundance. Diversity indices were determined using the diversity() function of the vegan package. Nonmetric multidimensional scaling for Bray-Curtis distance matrices were calculated using the metaMDS() function of the vegan package and principal coordinate decomposition of Weighted UniFrac distance matrices were calculated using the pcoa() function of the ape package (version 5.6.2). All analyses were performed on OTU level, genus level and family level. OTUs and genera contributing most to community differences between selected groups were extracted using the simper() function of the vegan package. Groupwise analysis of annotated reads was performed using RStudio (version 4.2.1) with an initial seed set at 8765. All operational taxonomic units (OTUs) not constituting at least 0.1% of reads within at least one group (group means) were removed from the analysis. Diversity indices were determined using the diversity() function of the vegan package (version 2.6.2). Nonmetric multidimensional scaling for Bray-Curtis distance matrices were calculated using the metaMDS() function of the vegan package and principal coordinate decomposition of weighted UniFrac distance matrices were calculated the pcoa() function of the ape package (version 5.6.2). All analyses were performed on OTU level, genus level and family level. OTUs and genera contributing most to community differences between selected groups were extracted using the simper() function of the vegan package.

Full-length 16S rRNA gene sequencing analysis:
Raw fast5 data files were converted to pod5 format using POD5 Tools (v0.2.0), then basecalled in super-accuracy mode and demultiplexed according to the barcodes of the SQK-NBD114-96 kit on a gpu partition using Dorado (v0.4.3). The basecalled and demultiplexed bam files for each sample have been uploaded to the European Nucleotide Archive (ENA) at EMBL-EBI under the study accession number PRJEB60278. Bam files were converted to fastq format using samtools (v1.16.1) bam2fastq, then filtered using NanoFilt (v2.8.0) such that only Phred quality scores above 10 and read lengths between 1,300 to 1,700 bp were retained. The taxonomic classification was carried out emu using emu (v3.4.5) with the --keep-counts flag and the default Emu 3.0+ database. Completely unassigned reads were removed from downstream analyses. The rarecurve() function from the vegan package (v2.6-4) was used to identify undersampled samples, which were also removed from downstream analyses. Count tables of the remaining samples were used to determine alpha-diversity using the phyloseq package (v1.40.4). To calculate beta-diversity and to perform further downstream analyses, only those detected bacterial features were included which provided more than 0.01% relative abundance in at least two mice within each group of microbiota-donor and microbiota-recipient combination. Beta-diversity measures were determined using the phyloseq package (v1.40.0) after read counts were normalized by calculating relative abundances followed by arcsine square root transformation. Relative abundances per taxonomic unit and sample were visualized using the fantaxtic package (v0.2.0).

ELISA analyses:
Concentrations were calculated based on detected optical densities (OD) of supplied standards and using R Studio (version 4.2.1) applying a 4-parameter nonlinear regression of standard ODs with the help of the drc package (version 3.0.1) using the function drm(OD~concentration, fct=LL.4()). Sample concentrations were then extracted using the ED(type=”absolute”) function of the same package.

Metatranscriptomics analyses:
RNA sequencing files were pre-processed using kneaddata (https://github.com/biobakery/kneaddata). Adapters were removed using Trimmomatic and fragments below 50% of the total expected read length (75 bp) were filtered out. BowTie2 was used to map and remove contaminant reads corresponding to either rRNA databases or the Mus musculus genome. Clean fastq files were concatenated before passing to HUMAnN3. A custom taxonomy table based on pooled 16S rRNA sequencing abundances was provided to MetaPhlAn (v30_CHOCOPhlAn_201901) for metagenome mapping. Reads were aligned within HUMAnN3 using bowtie2 (version 2.3). Unaligned reads were translated within HUMAnN3 using diamond (version 0.9.36) for protein identification using the UniRef90 database provided within HUMAnN3. Data for all samples were joined into a single table and normalized using count per million (CPM) method. Results were grouped by annotated protein product per individual. In case no annotation from UniRef90 transcript IDs was possible, distinct IDs were treated as separate gene products. Only gene products that provided >50 CPM in at least two of the eight investigated samples were included into downstream analyses. This resulted in 2213 transcripts being included into downstream analyses, representing 80% to 85% of the total CPM with no significant differences between the analyzed groups. CPM were recalculated to account for removed transcripts, followed by further analysis using the edgeR package (version 3.38.4) in R Studio (version 4.2.1). Multidimensional reduction of the transcriptome profiles was calculated using the logFC method within the plotMDS.DGEList function. Groupwise comparison of gene expression was calculated using the exactTest() function.

Metabolomics:
Metabolome spectra and concentration calculations were carried out using the software “MasterHands” version 2.19.0.2 (Keio University) as previously described (DOI: 10.1007/s11306-009-0178-y). Obtained cecal metabolite concentrations were converted from nmol × g−1 cecal content to pmol × g−1. Only the 175 cecal metabolites that were detectable in at least 50% of the samples within one of the seven tested groups were included in downstream analyses. Undetectable concentrations of these 175 metabolites in certain samples were replaced by a value of 1/3 of the lowest detectable value of this particular metabolite across all groups. Concentrations were then normalized using a log2-transformation for further downstream analysis.

For manuscripts utilizing custom algorithms or software that are central to the research but not yet described in published literature, software must be made available to editors and reviewers. We strongly encourage code deposition in a community repository (e.g. GitHub). See the Nature Portfolio guidelines for submitting code & software for further information.

Data

Policy information about availability of data

All manuscripts must include a data availability statement. This statement should provide the following information, where applicable:

- Accession codes, unique identifiers, or web links for publicly available datasets
- A description of any restrictions on data availability
- For clinical datasets or third party data, please ensure that the statement adheres to our policy

The raw files for this study from 16S rRNA gene sequencing (V4 and full-length amplicons) and RNA sequencing have been deposited in the European Nucleotide Archive (ENA) at EMBL-EBI under accession number PRJEB60278 (https://www.ebi.ac.uk/ena/browser/view/PRJEB60278). Raw spectral data from CE-TOF/MS metabolomic analyses are available on the MetaboBank integrated metabolome data repository under Project ID MTBKS223 (https://mb2.ddbj.nig.ac.jp/study/MTBKS223.html). For the construction of the phylogenetic tree of SM14 constituent strains, 16S rRNA gene sequences were downloaded from the ENA (https://www.ebi.ac.uk/ena/browser/home) using the following accession numbers: AY271254.1 (A. muciniphila), AB510697.1 (B. caccae), EU136682.1 (B. ovatus), HQ012026.1 (B. thetaiotaomicron), AB050110.1 (B. uniformis), AB370251.1 (B. intestinihominis), AB626630.1 (E. rectale), HM245954.1 (C. symbiosum), AJ505973.1 (M. formatexigens), AF192152.1 (D. piger), AB011816.1 (C. aerofaciens), AJ413954.1 (F. prausnitzii), AJ312385.1 (R. intestinalis), and CP000802.1:4065789..4067330:rRNA (E. coli). The Emu database used for full-length 16S rRNA gene mapping combines rrnDB v5.6 and NCBI 16S RefSeq from 17 September, 2020, and taxonomy from NCBI on the same date. Flow cytometry data is available on Zenodo (https://zenodo.org/records/12528901), under doi: 10.5281/zenodo.12528901.

Research involving human participants, their data, or biological material

Policy information about studies with human participants or human data. See also policy information about sex, gender (identity/presentation), and sexual orientation and race, ethnicity and racism.

Reporting on sex and gender	N/A
Reporting on race, ethnicity, or other socially relevant groupings	N/A
Population characteristics	N/A
Recruitment	N/A
Ethics oversight	N/A

Note that full information on the approval of the study protocol must also be provided in the manuscript.

Field-specific reporting

Please select the one below that is the best fit for your research. If you are not sure, read the appropriate sections before making your selection.

☒ Life sciences ☐ Behavioural & social sciences ☐ Ecological, evolutionary & environmental sciences

For a reference copy of the document with all sections, see nature.com/documents/nr-reporting-summary-flat.pdf

Life sciences study design

All studies must disclose on these points even when the disclosure is negative.

Sample size	We determined the sample sizes presented in the manuscript to be statistically sufficient to support the conclusions. See details on statistical calculation in the manuscript an in this report.
Data exclusions	For FACS analysis, low quality samples were excluded from analysis. "Low quality" was defined as "less than 1000 events after gating on CD45 + cells". Additionally, outliers were determined by group and values not within a range of mean ± 2σ were removed from the datasets.<br>For EAE-associated readouts, no data were excluded.<br>For groupwise comparisons of ELISA readouts, apparent outliers were identified using the outlier() function in R and confirmed by a Dixon-Test before being removed from the analysis.<br>In 16S rRNA gene sequencing analyses of SPF-housed mice, taxa not providing a mean relative abundance of 0.01% within a given group were excluded from downstream analysis. For RNA sequencing data, host RNA, ribosomal RNA and viral RNA were excluded from analyses. For metabolite analysis, metabolites not detected in at least 50% of the samples in at least 1 group were excluded from downstream analyses.
Replication	We performed EAE experiments in SPF-housed CR mice in 2 different runs. EAE experiments using SM14- and SM13-colonized mice were performed in 3 different runs (all attempts at replication were successful). As we observed the same internal group variances within each run, we determined that follow-up EAE experiments in other mice did not require multiple runs to cover the whole possible internal variance.
Randomization	Mice were assigned randomly to each group. After randomized group allocation, we checked whether the mean weight of each mouse group does not differ significantly from each other.

Blinding

Blinding was performed wherever possible. FACS analysis of isolated lymphocytes was performed blinded and the gating strategy was verified by at least two persons. EAE scoring, due to the nature of the experiment (e.g. visibly different diets, handling of mice from low to high SM to prevent contamination), complete blinding was impossible, however the EAE scoring was performed by two independent researchers (Alex Steimle and Mareike Neumann) to prevent bias. For CE-TOF/MS data generation, the researchers (Shinji Fukuda and Tomoyoshi Soga) were blinded for the identities of the experimental groups. For measurement of short-chain fatty acids from cecal samples, researchers from the responsible metabolomics facility were blinded for the identities of the experimental groups. For other group allocations during data collection and/or analysis, blinding was not possible or not needed for the following reasons: sequencing data (16S rRNA gene based and metatranscriptomics), blinding not needed as the same analysis pipeline was used and hence no bias expected; analysis of data from EAE scoring, blinding not needed as defined analysis criteria were employed and hence no bias expected; metabolomics data analysis, blinding not needed as defined analysis criteria were employed and hence no bias expected; bacterial IgA coating, blinding not needed as defined experimental protocols were employed and hence no bias expected; bacterial enzyme activities in stool samples, blinding not needed as defined experimental protocols were employed and hence no bias expected; ELISAs for LCN2, LPS, OCLN and ZO-1, blinding not needed as defined experimental protocols were employed and hence no bias expected.

Reporting for specific materials, systems and methods

We require information from authors about some types of materials, experimental systems and methods used in many studies. Here, indicate whether each material, system or method listed is relevant to your study. If you are not sure if a list item applies to your research, read the appropriate section before selecting a response.

Materials & experimental systems

n/a	Involved in the study
☐	☒ Antibodies
☒	☐ Eukaryotic cell lines
☒	☐ Palaeontology and archaeology
☐	☒ Animals and other organisms
☒	☐ Clinical data
☒	☐ Dual use research of concern
☒	☐ Plants

Methods

n/a	Involved in the study
☒	☐ ChIP-seq
☐	☒ Flow cytometry
☒	☐ MRI-based neuroimaging

Antibodies

Antibodies used

CD16/CD32, BD, #553142
rat anti-mouse IL-17A (TC11-18H10.1, 1/50, Biolegend, #506922)
rat anti-mouse RORγT (AFKJS-9, 1/44, eBioscience, #17-6988-82
rat anti-mouse CD3 (17A2, 1/88, Biolegend, #100241)
rat anti-mouse CD45 (30-F11, 1/88, BD, #564225)
rat anti-mouse CD4 (RM4-5, 1/700, Biolegend, #100548)
rat anti-mouse IFN-γ (XMG1.2, 1/175, eBioscience, #61-7311-82)
rat anti-mouse Foxp3 (FJK-16s, 1/200, eBioscience, #48-5773-82)
rat anti-mouse CD8 (53-6.7, 1/700, Biolegend, #100710)
rat anti-mouse Siglec-F (E50-2440, BD, #740956)
rat anti-mouse IgA (mA-6E1, 1/700, eBioscience, #11-4204-83)

Validation

Optimal concentrations for cell staining to exclude unspecific binding were determined in our lab using spleen cells. Antibody dilutions are indicated in the box above.
Faecal samples from Rag1−/− mice were used as non-IgA-coated negative controls in the determination of bacterial IgA coating indices.

Animals and other research organisms

Policy information about studies involving animals; ARRIVE guidelines recommended for reporting animal research, and Sex and Gender in Research

Laboratory animals

For gnotobiotic experiments, female germ-free (GF) C57BL/6N mice were used at the age of 5 to 8 weeks. The mice were originally purchased from Taconic Biosciences (USA) and were subsequently bred and housed in the GF facility of the University of Luxembourg, supervised by the Animal Experimentation Ethics Committee of the University of Luxembourg (AEEC).
For experiments performed under specific-pathogen-free (SPF) conditions, we used female mice of different origin. C57BL/6J wildtype mice were purchased from Charles River at the age of 5 to 8 weeks. Furthermore, we used mice lacking the Muc2 gene (strain designation: 129P2/OlaHsd×C57BL/6-Muc2<tm1Avel>), which were originally obtained from the lab of Kathy McCoy (University of Bern, Switzerland) under GF conditions. GF 129P2/OlaHsd×C57BL/6-Muc2<tm1Avel> mice were mated with SPF-housed C57BL/6J mice obtained from Charles River resulting in offspring heterozygous for presence of the Muc2 gene (Muc2+/−). Muc2+/− mice were constantly kept under the same SPF conditions as the SPF-housed parental C57BL/6J mice. Next, male and female Muc2+/− mice were mated and offspring were genotyped for absence and presence of the Muc2 gene. Homozygous Muc2−/− and Muc2+/+ mice obtained from this breeding were then used for experiments. Finally, faecal samples from SPF Rag1−/− mice (C57BL6/J background) at the Luxembourg Institute of Health were used as negative controls in IgA coating experiments.
Mice were housed in individually ventilated cages (Techniplast Sealsafe Plus GM500 or Allentown Sentry SPP™ Mouse cages in SPF or

GF facilities, respectively) at 20-24°C with 40-70% humidity, under 12-hour light cycles. Sterile water and diets were provided ad libitum.

Wild animals

No wild animals were used in the study.

Reporting on sex

The data reported in the manuscript was collected from female mice. We conducted preliminary experimental autoimmune encephalomyelitis (EAE) experiments on male gnotobiotic mice and found that male mice exhibited stronger EAE symptoms and could not be continued for the entire duration of the experiment owing to ethical constraints.
Generally, female individuals are more prone to the development of multiple sclerosis than male individuals, even though the progression of the disease is generally milder in females (DOI: https://doi.org/10.1177/1756285613488434). This is mainly due to the protective effect of female hormones and has been shown in mouse models of EAE (DOI: https://doi.org/10.1177/107385840100700310). Furthermore, male mice are more likely to be involved in territorial fights, which might influence the outcome of the experiments due to injuries. Due to these scientific facts and the results from the already performed experiments, we used only female mice.

Field-collected samples

No field collected samples were used in the study.

Ethics oversight

All mouse experiments followed a two-step animal protocol approval procedure. Protocols were first evaluated and pre-approved by either the Animal Experimentation Ethics Committee (AEEC) of the University of Luxembourg or the Animal Welfare System (AWS) of the Luxembourg Institute of Health, followed by final approval by the Luxembourgish Ministry of Agriculture, Viticulture, and Rural Development (Protocol numbers: LUPA2020/02, LUPA2020/27, LUPA2020/32, LUPA2019/43, LUPA2020/22, LUPA2019/51). All experiments were performed according to the Federation of European Laboratory Animal Science Association (FELASA). The study was conducted according to the “Règlement grand-ducal du 11 Janvier 2013 relatif à la protection des Animaux utilisés à des fins Scientifiques” based on the “Directive 2010/63/EU” of the European Parliament and the European Council from September 2010 on the protection of animals used for scientific purposes. All animals were exposed to 12 hours of light daily.

Note that full information on the approval of the study protocol must also be provided in the manuscript.

Flow Cytometry

Plots

Confirm that:

☒ The axis labels state the marker and fluorochrome used (e.g. CD4-FITC).

☒ The axis scales are clearly visible. Include numbers along axes only for bottom left plot of group (a 'group' is an analysis of identical markers).

☒ All plots are contour plots with outliers or pseudocolor plots.

☒ A numerical value for number of cells or percentage (with statistics) is provided.

Methodology

Sample preparation

Lymphocyte extraction from colonic lamina propria, ileal lamina propria and spinal cords:
After organ removal, lymphocytes from the colonic lamina propria (CLP), small intestine lamina propria (SILP) and spinal cords (SC) were extracted. While CLP and SILP lymphocytes were extracted using the lamina propria dissociation kit (Miltenyi Biotec, #130-097-410), SC lymphocytes were extracted using a brain dissociation kit (Miltenyi Biotec, #130-107-677), according to the manufacturer’s instructions. In brief, colon and ileum were dissected and stored in HBSS (w/o). Faeces and fat tissue were removed, organs were opened longitudinally, washed in HBSS (w/o), and cut laterally into 0.5 cm-long pieces. Tissue pieces were transferred into 20 mL of a predigestion solution (HBSS (w/o), 5 mM EDTA, 5% foetal bovine serum (FBS), 1 mM dithiothreitol) and kept for 20 min at 37 °C under continuous rotation. Samples were then vortexed for 10 sec and applied on a 100 µm cell strainer. Last two steps were repeated once. Tissue pieces were then transferred into HBSS (w/o) and kept for 20 min at 37 °C under continuous rotation. After vortexing for 10 sec, tissue pieces were applied on a 100 µm cell strainer. Tissue pieces were then transferred to a GentleMACS C Tube (Miltenyi Biotec, #130-093-237) containing 2.35 mL of a digestion solution and homogenized on a GentleMACS Octo Dissociator (Miltenyi Biotec, #130-096-427, program 37C_m_LPDK_1). Homogenates were resuspended in 5 mL PB Buffer (phosphate-buffered saline (PBS), pH 7.2, with 0.5% bovine serum albumin), passed through a 70 µm cell strainer and centrifuged at 300 × g for 10 min at 4 °C. Cell pellets were resuspended in ice-cold PB buffer and stored on ice until further use. Spinal cords were stored in ice-cold D-PBS until they were transferred to a GentleMACS C Tube containing a digestion solution. Samples were processed on a GentleMACS Octo Dissociator (program 37C_ABDK_01) and rinsed through a 70 µm cell strainer. The cell suspension flow through was then centrifuged at 300 × g for 10 min at 4 °C. Debris removal was performed by resuspending the cell pellet in 1550 µL D-PBS, adding 450 µL of Debris Removal Solution, and overlaying with 2 mL of D-PBS. Samples were centrifuged at 4 °C at 3,000 × g for 10 min. The two top phases were aspirated and the cell suspension was diluted with cold D-PBS. Samples were then inverted three times and centrifuged at 4 °C, 1,000 × g for 10 min and the cell were resuspended and stored in ice-cold D-PBS until further use.

Cell stimulation and flow cytometry:
10^6 cells (MLN cell suspensions as well as lymphocyte extracts from CLP, SILP and SC) were resuspended in 1 mL complete cell culture medium (RPMI containing 10% FBS, 2 mM glutamine, 50 U × mL-1 penicillin, 50 µg × mL-1 streptomycin, and 0.1% mercaptoethanol) supplemented with 2 µL Cell Activation Cocktail with Brefeldin A (Biolegend, #423304) and incubated for 4 h at 37 °C. Cells were centrifuged at 500 × g for 5 min, resuspended in 100 µL Zombie NIR (1:1000 in PBS, Zombie NIR™ Fixable Viability Kit, Biolegend, #423106), transferred into a 96-well plate and incubated for 20 min at 4 °C in the dark. Cells were washed two times with 150 µL PBS (centrifuged 5 min at 400 × g at 4 °C) and resuspended in 50 µL Fc-block (1:50, Purified Rat Anti-Mouse CD16/CD32, BD, #553142) diluted in FACS buffer (1x PBS/2% FBS/2mM, EDTA pH 8.0). Cells were

incubated for 20 min at 4 °C in the dark and washed two times with 150 µL PBS with centrifugation for 5 min at 400 × g at 4 °C. All cells were fixed for 30 min with BD Cytofix/Cytoperm solution (BD, #554722) and stored in PBS overnight. For the extracellular and intracellular fluorescent cell staining, cells were permeabilized with BD Perm/Wash buffer (BD, #554723) for 15 min. T lymphocytes were evaluated using the following antibodies: rat anti-mouse IL-17A (TC11-18H10.1, 1/50; Biolegend, #506922), rat anti-mouse RORγt (AFKJS-9, 1/44, eBioscience, #17-6988-82), rat anti-mouse CD3 (17A2, 1/88, Biolegend, #100241), rat anti-mouse CD45 (30-F11, 1/88, BD, #564225), rat anti-mouse CD4 (RM4-5, 1/700, Biolegend, #100548), rat anti-mouse IFN-γ (XMG1.2, 1/175, eBioscience, #61-7311-82), rat anti-mouse FOXP3 (FJK-16s, 1/200; ThermoFisher, #48-5773-82), rat anti-mouse CD8 (53-6.7, 1/700, Biolegend, #100710). Optimal staining concentrations of all antibodies were evaluated before staining. Cells were incubated with FACS buffer diluted antibodies for 30 min at 4 °C in the dark. Samples were washed twice with 150 µL of BD Perm/Wash buffer, resuspended in 200 µL PBS and acquired using NovoCyte Quanteon (NovoCyte Quanteon 4025, Agilent). All acquired data were analysed using FlowJo™ Software (version 10.7.2, BD, 2019). Fluorescence minus one controls (FMOs) were used for each antibody-fluorophore combination to properly evaluate signal-positive and -negative cells. Single antibody-stained UltraComp eBeads™ Compensation Beads (Fisher Scientific, Ref: 01-2222-42) were used to create the compensation matrix in FlowJo. Single antibody-stained compensation beads were created for each run separately, using the exact same antibody lot that were used for the samples. As we determined insufficient binding of the BV786-coupled rat anti-mouse CD45 antibody (30-F11, 1/88, BD, #564225) to the compensation beads, we used the BV786-coupled anti-mouse Siglec-F antibody (E50-2440, BD, #740956) to calculate the compensation matrix. Compensation samples were gated on the population of compensation beads within the FSC-H and SSC-H channels and the positive and negative population for the corresponding antibody were identified in a two-dimensional depiction of channels with strong fluorescence spillover. Compensation matrices were calculated for each run separately and applied to the samples of this particular run. After applying the compensation matrix, samples underwent the gating strategy, which is explained in detail in Extended Data Figure 7a and b. FACS analysis of isolated lymphocytes was performed blinded and the gating strategy was verified by at least two persons. Sample quality was evaluated by assessing the event distribution in the "SSC-H vs. FSC-H" and the "Live/Dead vs SSC-H" depiction and samples of insufficient quality and event counts were removed from the analysis. Downstream analysis of relative proportions of target cell populations was performed using RStudio (Version 4.2.1). Individual samples were grouped by target cell population, organ and EAE group phenotype (for non-EAE-induced mice) or by target cell population, organ and individual EAE severity cluster (for EAE-induced mice). Outliers were determined by group and values not within a range of mean ± 2σ were removed from the datasets.

Instrument

Quanteon NovoCyte (NovoCyte Quanteon 4025, Agilent)

Software

FlowJo™ Software (version 10.7.2, BD, 2019)

Cell population abundance

Cell population abundances are indicated for in Extended Data Figure 7 for all four organs of a representative EAE-induced (panel a) and all four organs of a representative non-EAE-induced (panel b) mouse. For the remaining mice, count data and proportions of each cell population as a percent of the defined parental population are provided in the Source Data for Extended Data Figure 7.

Gating strategy

The gating strategy to determine IL-17A and IFNγ expression in T-helper cells of EAE-induced and non-EAE-induced mice is depicted in Extended Data Figure 7 and described in the corresponding legend. To summarise:
(1) size selection of cells in SSC-H vs. FSC-H plot;
(2) selection of singlets in FSC-H vs. FSC-H plot;
(3) for MLN, CLP and SILP, but not SC, pre-gating step for CD45+ cells (not shown);
(4) selection of live, single cells in a Zombie NIR ("Live/Dead") vs. SSC-H plot;
(5) selection of single, live CD45+ cells in a CD3 vs. CD45 plot (for SC, gates were set according to the location of the CD45+ population in other organs of the same mouse due to auto-fluourescence of the SC cells);
(6) selection of T cells in a CD3 vs. SSC-H plot;
(7) selection of CD4+ T cells in a CD8 vs. CD4 plot;
(8) selection of IL-17A and IFNγ-expressing CD4+ T cells in an IFNγ vs. IL17A plot

☒ Tick this box to confirm that a figure exemplifying the gating strategy is provided in the Supplementary Information.
